# Technology-Based Obesity Prevention Interventions Among Hispanic Adolescents in the United States: Scoping Review

**DOI:** 10.2196/39261

**Published:** 2022-11-04

**Authors:** Erica G Soltero, Callie Lopez, Edith Hernandez, Teresia M O'Connor, Debbe Thompson

**Affiliations:** 1 United States Department of Agriculture/Agricultural Research Services Children’s Nutrition Research Center Department of Pediatrics Baylor College of Medicine Houston, TX United States

**Keywords:** obesity, technology, adolescents, health disparities, prevention interventions, prevention, intervention, feasibility, effectiveness, Hispanic, engagement

## Abstract

**Background:**

Given that today’s adolescents are digital front-runners, technology-based obesity prevention strategies are age-appropriate for this population. The use of remote and wireless technologies may be suitable for extending the reach and engagement of obesity prevention efforts among high-risk Hispanic youths, as this subgroup is disproportionately affected by barriers that limit participation in traditional, in-person interventions.

**Objective:**

The purpose of this scoping review was to examine the intervention and sample characteristics of technology-based obesity prevention interventions among Hispanic adolescents. We also examined feasibility criteria to assess the acceptability and appropriateness of technology-based strategies among Hispanic youths.

**Methods:**

A comprehensive search of Embase and PubMed identified 7 studies that met the inclusion criteria. Data were extracted by 2 independent reviewers.

**Results:**

Of the 7 included studies, half (n=4, 57%) used a randomized control trial design, with equal implementation in school (n=3, 43%) and clinic (n=4, 57%) settings. Studies commonly targeted improvements in diet (n=4, 57%) and physical activity (n=7, 100%), with only 1 (14%) study focused on sedentary behaviors. Just 2 (29%) studies reported the use of behavioral theories or models. Studies focused primarily on youths in early (n=5, 71%) or middle (n=6, 86%) adolescence, and there was limited information reported on socioeconomic status. Only 3 (43%) study conducted formative work, and few (n=3, 43%) reported on acceptability. Only 1 (14%) study reported that materials were available in Spanish and English, and only 1 (14%) study used culturally tailored content. Additionally, 3 (43%) studies used strategies that considered social determinants of health.

**Conclusions:**

To increase our understanding of the feasibility and effectiveness of technology-based obesity prevention strategies among Hispanic adolescents, there is a need for more feasibility studies that are theoretically grounded and comprehensively report on feasibility-related outcomes. Future studies should also leverage technology to simultaneously address multiple health behaviors beyond diet and physical activity. The result of this review can be used to guide the development of future technology-based obesity prevention strategies among Hispanic adolescents.

**Trial Registration:**

CliniclaTrials.gov NCT04953442; https://clinicaltrials.gov/ct2/show/NCT04953442

## Introduction

Lifestyle interventions that promote healthy diet and physical activity habits are the cornerstone for obesity prevention among adults and youths [1]. However, current lifestyle interventions have had a modest impact on reducing obesity and obesity-related behaviors among Hispanic youths [2-4]. For some Hispanic youths, the time-intensive nature of in-person interventions and the lack of studies that address negative social determinants of health (SDoH) can limit program participation and one’s ability to make healthy behavior changes [1,5]. SDoH that impact Hispanic youths include limitations in transportation, parent-work schedules, childcare needs, and access to health insurance, which can impact access to disease prevention opportunities in clinical settings [5,6]. Hispanic youths are disproportionately affected by obesity and obesity-related diseases and are the largest pediatric subgroup in the United States [7,8]. To address growing disparities, there is a substantial need to reach and engage this key population with obesity prevention strategies that are tailored to meet their needs and context [9].

Technology-based interventions use digital devices, such as computers, tablets, smartphones, and wearable devices, to deliver personalized and real-time health promotion and disease prevention interventions [10-13]. Given that Hispanic youths and families are disproportionately impacted by SDoH, the use of digital devices as behavior change tools has been suggested as a potential strategy for overcoming some of the negative SDoH that limit participation in traditional, in-person lifestyle interventions [14,15]. For example, web-based interventions are not confined by location and can be delivered across geographic regions directly to participants in their home environment, alleviating the burden of transportation [14]. Technology-based interventions can also offer flexible scheduling options or be continuously delivered using SMS text messaging, prerecorded video content, or eHealth apps, impacting the dose and timing in which an intervention can be delivered [14]. This flexibility may be helpful for engaging some Hispanic youths and families, given that many Hispanic parents have nontraditional working hours (eg, night shifts) or work more than 1 job, which can make attendance to in-person interventions challenging [16]. Technology-based strategies may also be cost-effective given that they leverage devices (ie, smartphones and tablets) and services (ie, SMS text messaging and social media) already owned and used by participants [17]. About 95% of Hispanic teens in the United States report that they have daily access to a smartphone, which is comparable to non-Hispanic White youths (94%), indicating that smartphones can be leveraged to reach this population [18]. However, despite their potential for overcoming barriers to in-person interventions, most technology-based interventions have been conducted among high-income populations [15,19]. Thus, there is a need for studies that are developed and tested among high-risk, vulnerable populations that are disproportionately impacted by these barriers [15,19].

Technology-based health promotion and disease prevention strategies are also recommended as being age-appropriate for adolescents [20]. Adolescents today are exposed to technology at a younger age and are digital front-runners [21]. Furthermore, nearly two-thirds of adolescents and young adults in the United States have reported using an app to support changes in diet or physical activity behaviors, suggesting a desire for technology-based behavioral strategies among this population [22]. Among adults, technology-based lifestyle interventions have led to significant improvements in weight-loss and the management of chronic diseases including type 2 diabetes and cardiovascular diseases [23-26]. However, the evidence base for the feasibility and efficacy of technology-based lifestyle interventions among adolescents is limited [10,27,28], and few studies have been tested among minority youths [19].

The purpose of this scoping review was to systematically examine the current state of the science on technology-based obesity prevention interventions among Hispanic adolescents. This review will provide descriptive information regarding the intervention and sample characteristics with a focus on feasibility criteria including formative work, measures of acceptability, and adaptations made for SDoH and cultural considerations. The focus on feasibility criteria will provide meaningful information on the appropriateness of technology-based intervention components and targets among Hispanic youths with obesity. Following an extensive review of the literature, we will summarize findings, identify knowledge gaps, and highlight next steps for future research among this population.

## Methods

This scoping review was conducted using the 5-stage methodological framework for scoping studies developed by Arksey and O’Malley [29]. In accordance with this framework, the steps used to complete the scoping review included (1) identifying the research question, (2) identifying relevant studies, (3) selecting studies, (4) charting the data, and (5) synthesizing and summarizing the results. The detailed methodology used to complete these steps are outlined below.

### Identifying the Research Questions

The following research questions guided this review: (1) What approaches were used to develop technology-based obesity prevention interventions among Hispanic adolescents? (2) What are the intervention and sample characteristics of technology-based prevention interventions implemented among Hispanic adolescents? and (3) Were outcomes regarding the feasibility of technology-based obesity prevention interventions among Hispanic youths reported in a feasibility or pilot study or earlier in the development of a fully powered trial? Given that few technology-based interventions have been developed for Hispanic youths, studies that have applied technology-based strategies among this population are primarily still in the pre-efficacy phase, and the feasibility of intervention components has yet to be confirmed [30]. To address this gap in the literature, we reviewed the current studies or previously published studies by the research team to evaluate outcomes related to the acceptability of the intervention, formative work conducted in the development phase, technical issues or barriers experienced, and any other factors that impacted the development or implementation of the intervention among this population [31].

### Identifying Relevant Studies

Studies identified as relevant to this scoping review were defined as empirical, peer-reviewed articles that described a technology-based obesity prevention intervention among Hispanic adolescents with obesity. A literature search of PubMed was conducted using a combination of the following Medical Subject Headings terms: obesity, adolescent, Hispanic, and intervention. The search strategy and combination of terms that were used are provided in Multimedia Appendix 1. This same search strategy was then applied to the Embase database. We reviewed the references for eligible articles; however, no other sources or search strategies were used to identify articles.

### Selecting Studies

Studies were selected using the following eligibility criteria: (1) included adolescents aged 13-18 years; (2) focused on obesity prevention or included a lifestyle intervention focused on reducing obesity outcomes (eg, weight, body mass index, and body fat) and cardiometabolic disease risk factors (eg, insulin, glucose, and cortisol); (3) written in English, (4) used a technology-based component; (5) conducted within the United States, given that Hispanic adolescents in this country have a unique sociocultural and environmental context; and (6) included a sample of at least 50% of participants who self-identify as Hispanic/Latino. This criterion has been used in previous reviews to ensure that studies are focused on Hispanic adolescents and that study findings are applicable to this population [32-34]. We did not have any criteria or limitations on publication date. Relevant studies were identified during the search and were screened first by the study title and then by the abstract using Endnote (Clarivate) referencing software. For the articles that met eligibility criteria based on title and abstract, the full article was assessed by 2 independent reviewers to confirm eligibility. Disagreements about study eligibility were discussed between the 2 reviewers and brought to a third party, when necessary, until a consensus was reached. There were no requirements for sample size, adolescent weight status, or study location. Articles were excluded if they (1) did not involve a technology-based component; (2) were protocol studies or nonintervention studies (eg, cross-sectional studies, qualitative studies, and review articles); or (3) were duplicates or had overlap with another study.

### Charting the Data

Once relevant articles were selected, information from all studies was extracted using a narrative review approach [35]. We developed an extraction framework that included 32 categories focused on information from the PRISMA-ScR (Preferred Reporting Items for Systematic reviews and Meta-Analyses extension for Scoping Reviews) checklist and our research questions. In all, 2 reviewers independently extracted information from each article across each data extraction category and met to compare the extracted information. Discrepancies were discussed between the 2 reviewers and a third party, when necessary, until a consensus was reached. The presence of available information across extraction categories is presented in Multimedia Appendix 2 [36-42].

### Synthesizing and Summarizing the Results

Descriptive statistics (ie, frequencies) were calculated for intervention and sample characteristics as well as feasibility-related components. A content analysis approach was used to summarize patterns found in the information extracted across data extraction categories [29]. Data synthesis and summation was focused on answering the research questions.

## Results

### Database Search and Screening

**Figure 1:**
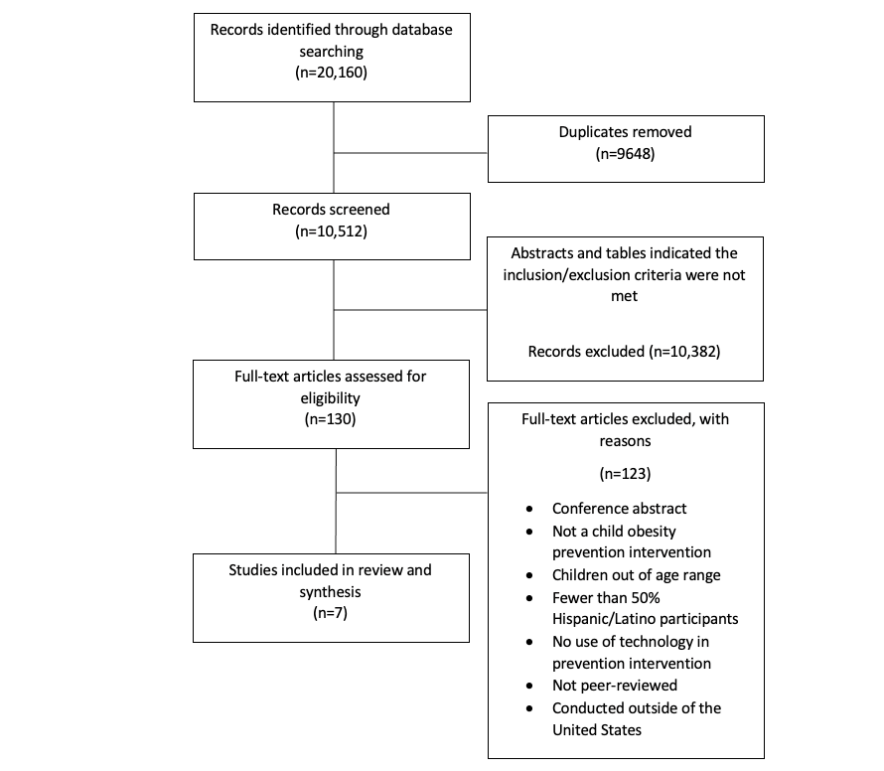
Consolidated Standards of Reporting Trials (CONSORT) diagram.

The search yielded a total of 20,160 results, with 10,512 remaining after duplicates were removed. The Consolidated Standards of Reporting Trials (CONSORT) diagram in Figure 1 summarizes the review process. A total of 10,382 papers were eliminated by the blind screening of titles and abstracts. After reading the full article, 123 papers were eliminated for the following reasons: being a conference abstract, not being an adolescent obesity prevention intervention, adolescents were out of the age range, conducted outside of the United States, included fewer than 50% Hispanic/Latino participants, no use of technology in prevention intervention, and not being a peer-reviewed paper. The search yielded a total of 7 papers published between 2010-2021 that were included in this scoping review.

### Intervention Characteristics

Table 1 provides a summary of intervention characteristics. Of the 7 included papers, half (n=4, 57%) of all studies were randomized control trials, with the other half (n=3, 43%) representing quasi-experimental study designs including pre-post feasibility and pilot studies. There were about equal numbers of interventions that were implemented in the school (n=3, 43%) and clinic (n=4, 57%) settings. Additionally, 2 interventions implemented in the school setting and 2 interventions implemented in the clinic setting also had a home-based component. Most (n=5, 71%) interventions were fewer than or equal to 12 weeks in duration, with a few (n=2, 29%) lasting from 11-52 weeks, and no study lasting longer than 1 year. Regarding obesity-related health behaviors, all (n=7, 100%) interventions targeted physical activity and most (n=4, 57%) focused on dietary habits. Most (n=5, 71%) studies did not report the use of a theoretical framework. For the studies that did, they reported the use of multiple theories including the following: Transtheoretical Model of Change, Mindset Theory, Achievement Motivation Theory, and Behavioral Determinants Model. Web-based sessions were the most (n=4, 57%) commonly used technology-based approach sessions, typically lasted 30-45 minutes. All (n=7, 100%) the studies reviewed described the use of at least one behavior change techniques, with many studies using more than one. Enhancing social support (n=5, 71%) and self-efficacy (n=6, 86%) were the most commonly used techniques, followed by the use of didactic health education sessions (n=5, 71%). Regarding primary outcomes, all (n=7, 100%) studies assessed physical activity with over half of the studies also assessing diet (n=4, 57%), anthropometrics or cardiometabolic outcomes (n=6, 86%), and a psychosocial outcome (n=5, 71%). We found 4 (57%) studies that reported significant improvements in health behaviors, 3 (43%) studies that reported improvements in obesity or cardiometabolic disease outcomes, and 3 (43%) studies that reported improvements to psychosocial outcomes.

**Table 1:** Intervention characteristics.

Characteristic		Study (N=7), n (%)
**Study design^a^**		
	Randomized controlled trial	4 (57)
	Quasi-experimental	3 (43)
**Intervention setting^a^**		
	School	3 (43)
	Clinic	4 (57)
	Home	4 (57)
**Length of intervention**		
	≤12 weeks	5 (71)
	13 weeks to 1 year	2 (29)
**Health behaviors targeted^a^**		
	Dietary habits	4 (57)
	Physical activity	7 (100)
	Weight loss or regulation	2 (29)
	Sedentary behaviors	1 (14)
**Theoretical framework^a^**		
	Transtheoretical Model of Behavior Change	1 (14)
	Mindset Theory	1 (14)
	Achievement Motivation Theory	1 (14)
	Behavioral Determinants Model	1 (14)
	Not specified	5 (71)
**Technology components used^a^**		
	Web-based sessions	4 (57)
	Fitness tracker or pedometer	2 (29)
	Telephone-based	2 (29)
	SMS text messaging	1 (14)
	Heart rate monitor	1 (14)
	Video gaming system	1 (14)
**Behavior change techniques^a^**		
	Social support	5 (71)
	Promoting self-efficacy	6 (86)
	Behavioral counseling	1 (14)
	Stop light approach	1 (14)
	Self-monitoring	1 (14)
	Health education	5 (71)
**Primary outcomes^a^**		
	Anthropometrics	6 (86)
	Diet	4 (57)
	Physical activity	7 (100)
	Sedentary behaviors	1 (14)
	Screen time	1 (14)
	Biomarkers	2 (29)
	Fitness	2 (29)
	Psychosocial outcomes	5 (71)

**^a^**Indicates that categories are not mutually exclusive, and total may exceed 100%.

### Sample Characteristics

Participant characteristics are presented in Table 2. Almost all interventions included an overlapping population of youths in early and middle adolescence aged 10-13 years (n=5, 71%) and 14-17 years (n=6, 86%), respectively, with just 1 (14%) study including older adolescents aged 18-21 years. Sample sizes varied, with most (n=6, 86%) studies having 200 participants or fewer, and just 1 (14%) study having over 300 participants. Most (n=5, 71%) studies did not present data on family socioeconomic status. Among studies that did (n=2, 29%), they focused on youths from lower socioeconomic backgrounds. Most (n=6, 86%) interventions were designed to engage adolescents only; however, 1 (14%) study focused on both the parent and adolescent.

**Table 2:** Sample characteristics.

Characteristic		Study (N=7), n (%)
**Age^a^**		
	Early adolescence (10-13 years)	5 (71)
	Middle adolescence (14-17 years)	6 (86)
	Late adolescence (18-21 years)	1 (14)
**Sample size**		
	0-100	4 (57)
	101-200	2 (29)
	>300	1 (14)
**Family socioeconomic status**		
	Low socioeconomic status	2 (29)
	Not specified	5 (71)
**Program participant**		
	Youths and family	1 (14)
	Youths only	6 (86)

**^a^**Indicates that categories are not mutually exclusive, and total may exceed 100%.

### Feasibility-Related Criteria

Feasibility-related characteristics are presented in Table 3. Only 3 (43%) studies conducted formative work. Formative work included pilot-testing intervention strategies [36], usability testing [37], and qualitative focus groups to guide intervention development [38]. The formative work conducted yielded information on the technical issues, level of participant engagement, and age-appropriateness of technology-based components [36-38]. Additionally, 1 (14%) study was delivered simultaneously in Spanish and English [36]. No other study specified the language used (n=6, 86%). There was also no study that reported the use of culturally tailored content in their intervention. Regarding SDoH, only a few (n=3, 43%) studies addressed or considered SDoH that were barriers in their development or implementation phase of the intervention. These strategies included identifying perceived self-reported barriers to physical activity [39], delivering the intervention on the web to overcome barriers such as transportation [40], and collaborating with community clinics and conducting provider trainings to focus on high-risk patients [36]. Only a few (n=3, 43%) studies included a measure of acceptability [38-40]. These studies used qualitative interviews, focus groups, and a postintervention satisfaction survey to measure acceptability. Flynn et al [39] reported 90% enjoyment among participants; Weigensberg et al [38] rated enjoyment on a scale from 1 to 10, and all participants reported scores of 9-10; whereas Jones et al [40] reported high satisfaction; however, only survey findings were presented. Only 2 (29%) studies reported on technical issues, which included device malfunctioning [41] and technical issues with computers in the school setting [40]. Finally, a few (n=3, 42%) studies reported retention rates above 80%, with a few (n=2, 29%) studies reporting retention rates below 80%, including Bowen-Jallow et al [41] (54.2%) and Patrick et al [36] (63%).

**Table 3:** Feasibility-related characteristics.

Characteristic		Study (N=7), n (%)
**Formative work**		
	Yes	3 (43)
	Not specified	4 (57)
**Language**		
	Bilingual	1 (14)
	Not specified	6 (86)
**Culturally tailored content**		
	Not specified	7 (100)
**Acknowledged social determinants of health**		
	Yes	3 (43)
	Not specified	4 (57)
**Acceptability measure**		
	Yes	3 (43)
	Not specified	4 (57)
**Technology issues reported**		
	Yes	2 (29)
	Not specified	5 (71)
**Retention rates**		
	0%-80%	2 (29)
	81%-90%	3 (43)
	Not specified	2 (29)

## Discussion

### Principal Findings

Technology-based interventions are a promising, age-appropriate, and accessible approach for engaging high-risk youths in disease prevention efforts; however, few such interventions have been developed and tested specifically for Hispanic youths. This scoping review used rigorous methods to review technology-based obesity prevention interventions among Hispanic adolescents. This review examined intervention and sample characteristics. Strengths in intervention and sample characteristics include the use of a rigorous randomized controlled trial study designs among half of all studies, although only 2 reported that they were fully powered. Most studies assessed physical activity, diet, anthropometrics, cardiometabolic biomarkers, and a psychosocial outcome. Although the focus of a scoping review is not on study outcomes, it is worth noting that studies that reported significant improvements to anthropometric or cardiometabolic outcomes used a hybrid approach of in-person and remote technology strategies and reported high acceptability and retention (≥80%) [38,40,42]. Although it is not clear what the most effective behavior change techniques are for Hispanic youths given the broad range of techniques used, all studies reported the use of 1 or more behavior change technique, which is a noteworthy strength [43,44].

This review also focused on the reporting of feasibility-related outcomes in each study. Only a few studies reported on technical issues, and it is not clear if this is because few technical issues were experienced or if the investigators did not publish this information. Of the studies that did publish on acceptability, they reported very high levels of satisfaction and enjoyment. Attrition, another indicator of engagement and feasibility, was mixed, with some studies reporting high levels of attrition. Taken together, reporting on feasibility criteria across studies in this review are limited, and although the technology-based strategies used in these interventions are promising, there is a greater need for the testing and publishing of feasibility-related criteria. To increase the feasibility, reach, and begin to move toward efficacy, there are substantial gaps that future technology-based prevention strategies should address.

### Identified Gaps and Implications for Future Research

#### Lack of Theoretical Framework

Only 2 studies in this review reported the use of behavioral theories and models [36,37]. These 2 studies reported the use and integration of multiple theories; however, neither study assessed theoretical constructs nor examined them as mediators of intervention effects [36,37]. Given this gap in reporting, there is limited information on the theoretical constructs that drive behavior change in technology-based interventions among this population [1,45,46]. It has been suggested that technology-based interventions require new, adaptable theoretical approaches that build upon existing behavioral theories to integrate the design, implementation, and engineering needs of technology-based strategies [47,48]. Among racial and ethnic subgroups, theoretical approaches should also address the social and cultural needs of the population of focus [45]. To advance the state of the science in technology-based interventions among Hispanic youths, there is a need for more theoretically grounded interventions. Future studies should provide more detail on the theoretical approaches used and any adaptations that are made. This information is critical for identifying and understanding the underlying theoretical mechanisms by which these interventions drive behavior change and reduce obesity among Hispanic youths [49,50].

#### Lack of Reporting on Feasibility Criteria

To assess the acceptability and appropriateness of technology-based strategies among Hispanic adolescents, we examined the reporting of feasibility-related criteria including formative work, measures of acceptability, and adaptations for SDoH or cultural considerations. Just half of studies conducted formative work [36-38], which is consistent with previous reports that youths are often not included in the development of technology-based interventions [20]. User-centered or co-design approaches that engage the end user in the design and development process can significantly increase the acceptability, engagement, and effectiveness of technology-based strategies [28]. Only 3 studies included measures of acceptability, limiting our understanding of the age and cultural appropriateness of the strategies used. Similarly, just 3 studies acknowledged SDoH. Hispanic youths are disproportionately burdened by inequitable experiences across obesity-related SDoH [51]. This finding underscores the need to address negative SDoH such as the lack of transportation as well as seek opportunities to leverage positive SDoH such as family social support and connectedness in the design and implementation of prevention efforts [52]. Regarding cultural considerations, just 1 study reported that they offered materials to participants in Spanish and English in consideration of language barriers [36]. Although peripheral strategies such as language translations are needed [9], there is also a need for more “deep structure” strategies that integrate broader social and cultural factors such as values, norms, and traditions [53]. Interventions that are culturally tailored to the focus population are the most effective and engaging interventions for addressing obesity disparities among minority youths [9,32,54]. Studies in this review also had limited reporting on other feasibility-related criteria including technical issues experienced by implementers or participants as well as the socioeconomic makeup of participants. These findings suggest that current technology-based interventions are not adapted to the cultural and social context of Hispanic adolescents. Furthermore, these findings highlight the substantial need for increased reporting on feasibility-related outcomes to discern if technology-based strategies are engaging and appropriate for high-risk youths and the barriers they may face [1,20].

#### Limitations in Health Behaviors Targeted

Lastly, similar to previously published reviews of obesity prevention interventions [32,46], we found that studies focused narrowly on diet and physical activity, with only 1 study that targeted sedentary behaviors [40] and no study focused on sleep behaviors. Time spent in sedentary pursuits, including screen time, is associated with higher BMI and poor lifestyle behaviors including increased caloric consumption and reduced activity [55]. Hispanic adolescents, particularly those from low-income households, engage in more screen time compared to non-Hispanic White youths [56], highlighting the importance for technology-based interventions among this population to address sedentary behaviors [1]. It has also been suggested that investigators specifically address screen time given that technology-based strategies may be seen as promoting screen time or as contrary to screen time recommendations [1]. Hispanic adolescents also report lower amounts of sleep compared to non-Hispanic White adolescents, and insufficient sleep is associated with greater risk for obesity [56]. Many technology devices such as personal activity trackers and some smartphone apps are already designed to promote and collect data on wake-time activity and nighttime sleep behaviors [57]. The 24-hour activity and sleep paradigm holds that to increase the effectiveness of current obesity prevention efforts, future interventions should leverage these devices to address the full continuum of wake-time activity and sleep behaviors [58].

### Strengths and Limitations

This study focused on a high-risk population that is traditionally underrepresented in research. This review will contribute to the limited body of research describing technology-based obesity prevention interventions among Hispanic youths with obesity. Additional strengths included a rigorous, comprehensive search strategy across numerous databases and a systematic, in-depth data extraction process that was performed in duplicate to ensure reliability. Some studies may have had information missing across data extraction categories (ie, examined theoretical mediators) given that they were feasibility and pilot studies. We did not assess intervention effectiveness or quality, which may be seen as a limitation; however, a more rigorous assessment of outcomes is more in line with a systematic review and not a scoping review. Lastly, the results of this study may be influenced by the search terms that were used, the use of US-based search engines, the number of databases searched, the focus on English-language articles, and the selection of databases used in the search. As a result, this review may be subject to publication bias.

### Conclusions

The literature on technology-based obesity prevention efforts among Hispanic adolescents is limited, making it difficult to determine the feasibility of this promising approach among this population. In addition to greater testing and reporting on feasibility-related outcomes, this review highlights 3 key gaps that should be addressed in future studies. There is a need for technology-based obesity prevention interventions that are theoretically grounded and that evaluate theoretical constructs to identify the underlying mechanism by which these strategies impact obesity-related outcomes and health behaviors among high-risk youths. There is a need for interventions that are tailored to the context of Hispanic youths and a need for increased evaluation and reporting of feasibility-related outcomes of these interventions to determine the acceptability and appropriateness of technology-based strategies for Hispanic youths. Furthermore, given known disparities in screen time and sleep among Hispanic youths, intervention strategies among this population should leverage technology to address a broader range of health behaviors, including sedentary behaviors and sleep, to increase program effectiveness. Addressing these gaps in future work will guide the development and implementation of technology-based obesity prevention efforts that aim to reduce obesity disparities and promote health equity among Hispanic adolescents.

## Appendix

Multimedia Appendix 1 Search strategy used to identify eligible technology-based obesity prevention interventions among Hispanic adolescents with obesity.

Multimedia Appendix 2 Data extraction categories and availability of data within each article included in the review (n=7).

## Abbreviations

CONSORT: Consolidated Standards of Reporting Trials
PRISMA-ScR: Preferred Reporting Items for Systematic reviews and Meta-Analyses extension for Scoping Reviews
SDoH: social determinants of health
