# Supplementary material for: Technology-Based Obesity Prevention Interventions Among Hispanic Adolescents in the United States: Scoping Review
Source: JMIR Pediatr Parent. 2022 Nov 4;5(4):e39261. doi: 10.2196/39261 (PMC9675012; doi:10.2196/39261)
Supplement: Multimedia Appendix 1 [file pediatrics_v5i4e39261_app1.docx]

**Table S1.** Search strategy used to identify eligible technology-based obesity prevention interventions among Hispanic adolescents with obesity.

"obesity" [Mesh] OR "pediatric obesity" [Mesh] OR "overweight" [Mesh]

AND

"adolescents" [Mesh] OR "teens" [Mesh] OR "adolescent" [Mesh]

AND

"intervention" OR "prevention" OR "program"

AND

"mhealth" [Mesh] OR "mhealth app" [Mesh] OR "text message" [Mesh] OR "website" [Mesh] OR "computer" [Mesh] OR "mobile phone" [Mesh] OR "health app" [Mesh] OR "ehealth" [Mesh] OR "online" [Mesh] OR "wearable" [Mesh] OR "wearable technology" [Mesh] OR "physical activity tracker" [Mesh] OR "telehealth" [Mesh] OR "telemedicine" [Mesh] OR "digital" [Mesh] OR "video game" [Mesh] OR "computer game" [Mesh] OR "social media" [Mesh] OR "technology" [Mesh]

AND

“Latino” [Mesh] OR “Spanish” [Mesh] OR “Hispanic”
