# Supplementary material for: Technology-Based Obesity Prevention Interventions Among Hispanic Adolescents in the United States: Scoping Review
Source: JMIR Pediatr Parent. 2022 Nov 4;5(4):e39261. doi: 10.2196/39261 (PMC9675012; doi:10.2196/39261)
Supplement: Multimedia Appendix 2 [file pediatrics_v5i4e39261_app2.docx]

**Table S2**. Data extraction categories and availability of data within each article included in the review (n=7).

| Author (year) | Study Design | Intervention  Length | Ethnicity | SES^1^ | Sample Size | Geographic Location | Theoretical Framework | Acceptability  Measure | Strategies to address SDoH | Technical Issues | Retention % |
| --- | --- | --- | --- | --- | --- | --- | --- | --- | --- | --- | --- |
| Bowen-Jallow et al [41] (2021) | √ | √ | √ |  | √ | √ |  |  |  | √ | √ |
| Flynn et al [39] (2018) | √ | √ | √ | √ | √ | √ |  | √ | √ |  |  |
| Garza et al [42] (2019) | √ | √ | √ | √ | √ | √ |  |  |  |  | √ |
| Jones et al [40] (2014) | √ | √ | √ |  | √ | √ |  | √ | √ | √ | √ |
| Weigensberg et al [38] (2014) | √ | √ | √ |  | √ | √ |  | √ |  |  | √ |
| Orvidas et al [37] (2020) | √ | √ | √ |  | √ | √ | √ |  |  |  |  |
| Patrick et al [36] (2013) | √ | √ | √ |  | √ | √ | √ |  | √ |  | √ |

^1^SES= Socioeconomic status

**Table S2 (Cont).** Data extraction categories and availability of data within each article included in the review (n=7).

| Author (year) | Behaviors Promoted | Behavior  Change  Techniques | Program Implementer | Language | Setting | Outcomes  /Results | Technology Components  Used | Culturally Tailored | Formative Work | Intervention Recipients | |
| --- | --- | --- | --- | --- | --- | --- | --- | --- | --- | --- | --- |
| Bowen-Jallow et al [41] (2021) | √ | √ | √ |  | √ | √ | √ |  |  | √ |  |
| Flynn et al [39] (2018) | √ | √ | √ |  | √ | √ | √ |  |  | √ |  |
| Garza et al [42] (2019) | √ | √ | √ |  | √ | √ | √ |  |  | √ |  |
| Jones et al [40] (2014) | √ | √ | √ |  | √ | √ | √ |  |  | √ |  |
| Weigensberg et al [38] (2014) | √ | √ | √ |  | √ | √ | √ |  | √ | √ |  |
| Orvidas et al [37] (2020) | √ | √ | √ |  | √ | √ | √ |  | √ | √ |  |
| Patrick et al [36] (2013) | √ | √ | √ | √ | √ | √ | √ |  | √ | √ |  |
